# In vitro *s*creening of known drugs identified by scaffold hopping techniques shows promising leishmanicidal activity for suramin and netilmicin

**DOI:** 10.1186/s13104-018-3446-y

**Published:** 2018-05-21

**Authors:** Supriya Khanra, Y. Pavan Kumar, Jyotirmayee Dash, Rahul Banerjee

**Affiliations:** 1 Crystallography and Molecular Biology Division, Saha Institute of Nuclear Physics, Sector 1, Block AF, Bidhannagar, Kolkata, 700064 India; 2 Homi Bhabha National Institute, Anushakti Nagar, Mumbai, 400094 India; 3 Department of Organic Chemistry, Indian Association for the Cultivation of Science, Jadavpur, Kolkata, 700032 India

**Keywords:** Netilmicin, Curcumin, Suramin, Leishmaniasis, Synergism

## Abstract

**Objective:**

The rapid emergence of drug resistant *Leishmanial* strains makes it imperative to continue the development of cheap and effective drugs against the parasite. Due to the absence of effective vaccines against leishmaniasis, current therapeutic measures exclusively rely on chemotherapy. Here we attempt, to identify novel antileishmanial from a list of known drugs determined from a previous bioinformatics study. Synergism between various drug combinations (involving netilmicin, suramin, paromomycin and curcumin) have been estimated to identify potent multidrug therapies to combat the disease.

**Results:**

The drugs were screened against *Leishmania* promastigotes by utilizing the MTT assay and against intracellular amastigotes using murine Macrophage like tumor cell, RAW 264.7 as a host. In vitro drug interactions were tested for several drug combinations with a modified fixed ratio isobologram method against both *Leishmania major* and *Leishmania donovani*. This work reports the in vitro antileishmanial activity for the aminoglycoside netilmicin (for some *Leishmania* parasites) and the anti-trypanosomatid suramin. Synergism was also observed between paromomycin–suramin and netilmicin–curcumin.

**Electronic supplementary material:**

The online version of this article (10.1186/s13104-018-3446-y) contains supplementary material, which is available to authorized users.

## Introduction

Leishmaniasis, a broad spectrum of neglected tropical diseases caused by the protozoan parasites *Leishmania* spp., exhibits a wide variety of clinical symptoms, epidemiology and pathogenesis [1]. Traditionally, leishmaniasis is classified into three different clinical manifestations: cutaneous (CL), mucocutaneous (MCL) and visceral (VL) or kala-azar (KA). Approximately 20 million people are infected with leishmaniasis worldwide [2]. VL is endemic in the Indian subcontinent and expanding its base on the Gangetic plains of Bangladesh, India and Nepal [3]. East Africa is second only to India in the incidence of VL and highest in HIV–VL co-infection rate [4]. Over a span of 30 years, VL has escalated from rural areas to urban centers in Brazil, spreading across the whole country [5]. The highest incidence of CL is in Afghanistan with an estimated 200,000 reported cases per year from Kabul alone [6].

Several *Leishmanial* strains responsible for VL in the Indian subcontinent have been reported to be resistant to antimonial drugs, the traditional first line of defense against the parasite [7, 8]. Other drugs used as a replacement for antimonials include amphotericin B, pentamidine, miltefosine and paromomycin some of which are expensive, difficult to administer and exhibit severe side effects [9, 10]. Of these, resistant strains have already been reported for amphotericin B and miltefosine [11, 12]. The above scenario indicates that the search for economically viable antileishmanial agents of reduced toxicity must remain unabated. In keeping with this challenge several prospective novel antiparasitic compounds have also been reported [13–16].

Previous work in the laboratory reported a bioinformatics study identifying drugs by scaffold hopping techniques, which could be prospective antileishmanials [17]. The two objectives in this work, are firstly to experimentally screen a subset of these compounds, selected from the final list of 32 approved drugs (identified by bioinformatics) for antileishmanial activity (Additional file 1). The compounds curcumin and suramin have been included in the screening as both have been reported for antileishmanial [18] and antitrypanosomal [19] activity respectively. Secondly, the synergism between several drugs has been tested with a view to formulate potent and effective therapeutic remedies for possible use in combination therapy.

## Main text

### Methods

#### *Leishmania* parasites culture

*Leishmania major* (5ASKH), *L. donovani* (MHOM/IN/83/AG83) and *L. donovani* (BS13) promastigotes were routinely cultured at 22 °C in M199 medium (St. Louis, MO, USA) with 10% heat-inactivated Fetal Bovine Serum (FBS) (Gibco, USA). Drug sensitivity was assayed using MTT and Giemsa stain in the amastigote–macrophage model, with each experiment being performed in triplicate. The final list of drugs reported in this study are paromomycin, suramin, primaquine, curcumin (all from Sigma-Aldrich Ltd.) and netilmicin (Zuventus Healthcare Ltd).

#### Cell line used for in vitro study

Murine Macrophage (MØs) like tumor cell, RAW 264.7 was obtained from American Type Culture Collection and were maintained in complete RPMI 1640 medium (HiMedia) with 10% FBS at 37 °C with 5% CO_2_ in a humidified atmosphere.

#### Determination of efficacy of the studied compounds on *L. major* promastigotes (IC_50_)

Day 5 culture of *L. major* promastigotes were used to determine the drug efficacy (IC_50_) using the MTT assay [20]. Briefly, *L. major* parasites were plated on 96-well cell culture plates at a density of 10^5^ parasites/well and incubated with different concentrations of the respective drug solutions for 72 h. The concentration which inhibited parasitic growth by 50% (IC_50_) was determined using the GraphPad Prism 5 software (version 5.03) [21] and the same software was utilized to estimate the statistical significance of drug effectiveness by one way analysis of variance (ANOVA). P value of < 0.05 was considered to be significant in terms of drug efficacy.

#### In vitro drug susceptibility assay against intracellular *L. major* and *L. donovani* amastigotes

The drug susceptibility of *Leishmania* amastigotes was assessed as described previously [22]. Briefly, the murine macrophage (MØs) like tumor cell, RAW 264.7 were allowed to adhere to the experimental cover slips for 24 h at 37 °C under 5% CO_2_. The adherent macrophages (MØs) were then infected either with *L. donovani* or with *L. major* promastigotes at a ratio of 1:10 (MØs: parasites) respectively and incubated further for 6 h at 37 °C under 5% CO_2_. After 6 h, excess parasites were removed by washing with serum-free medium. This was considered to be the initial time point of infection (0 h) and the infection was allowed to progress overnight [22]. Subsequently, the infected cells were incubated with different concentrations of the drug solutions. Untreated macrophages which served as controls received RPMI complete medium and further processing of the infected macrophages commenced after 48 h.

#### Estimation of EC_50_ value

The experimental cover slips consisting of infected MØs were washed with sterile PBS, dried, fixed with 100% methanol, stained with 15% Giemsa (Sigma) and examined under microscope. The amastigotes were counted and scored based on 100 MØs/cover slips. The values of the half maximal effective concentration (EC_50_) for the drugs were calculated for intracellular amastigotes.

#### Evaluation of drug interactions on the growth of *L. major* promastigote and construction of isobologram

The “modified fixed-ratio isobologram” method was used to determine the nature of drug interaction in its effect on *L. major* promastigote growth [23] by the MTT assay. Briefly, fixed-ratio solutions consisting of two drugs, at ratios 5:0, 4:1, 3:2, 2:3, 1:4 and 0:5 were prepared for all the possible drug combinations (involving paromomycin, primaquine, netilmicin, suramin) and each such fixed ratio solution was serially diluted six times in twofold dilutions. Predetermined IC_50_ values were used to decide the maximum concentrations of the individual drugs to ensure that the respective IC_50_’s were located near the midpoint of the six point twofold dilutions series. Using the procedures described above the IC_50_ values were again determined for promastigotes, subsequent to an exposure of 72 h to the solutions consisting of the appropriate drug combinations [24]. The fractional inhibitory concentrations (FICs) were calculated as described by Berenbaum [25] and defined as:

$${\mathbf{FIC}} = \, \left( {{\mathbf{IC}}_{{{\mathbf{50}}}} } \right)_{{{\mathbf{XY}}}} /\left( {{\mathbf{IC}}_{{{\mathbf{50}}}} } \right)_{{\mathbf{X}}}$$

where (IC_50_)_X_ is the IC_50_ value for drug X acting alone and (IC_50_)_XY_ is the IC_50_ for the same drug in the presence of a suboptimal concentration of drug Y. FICs and sum FICs ($\sum {\text{FICs}}$ [FIC for drug X + FIC for drug Y]) were calculated for all fixed-ratio solutions. FICs were used to construct classical isobolograms [26] and the mean of $\sum {\text{FICs}}$ were used to define the extent of synergism between the drugs. Presence of synergy was indicated by $\sum {\text{FIC}}$ ≤ 0.5; indifference or additive (4 ≥  $\sum {\text{FIC}}$ > 0.5); whereas antagonism $\sum {\text{FIC}}$ of > 4 [27].

#### In vitro assessment of drug interactions for intracellular amastigotes

In vitro drug interactions for intracellular *L. major* and *L. donovani* amastigotes were assessed by the “modified fixed-ratio isobologram method” [23] using the amastigote–macrophage model. Possible drug combinations were selected on the basis of their previously determined interactions against promastigotes and their efficacy against intracellular amastigotes. Drug activity was determined from the percentage of infected macrophages after treatment in relation to the non-treated infected macrophages after methanol fixation and Giemsa staining of the experimental cover slips. EC_50_ were then determined on intracellular amastigotes, subsequent to an exposure of 48 h to solutions consisting of appropriate drug combinations. Then FICs were calculated to construct classical isobolograms [24, 25].

### Results

#### Drug screening against *Leishmania* promastigotes and amastigotes

Drugs which were not lethal to the parasite even at concentrations in excess of 1500 µM were considered ineffective (NE) whereas all drugs whose IC_50_ values exceeded 400 µM were not considered for further experiments (Additional file 1).

No antileishmanial activity was found for the drugs triamterene (P = 0.0891), vidarabine (P = 0.1347), kanamycin (P = 0.0703), tobramycin (P = 0.1222), framycetin (P = 0.0836), lidocaine (P = 0.0632) whereas for the drugs acarbose and gentamicin, the respective IC_50_ values were in excess of 400 μM. These drugs were excluded from further downstream experiments. Paromomycin whose IC_50_ (50 ± 2.5 μM for *L. donovani* promastigotes) had been previously reported [28] was used as a control for all experiments. In case of *L. major* promastigotes the IC_50_ for paromomycin was determined to be 40.8 ± 3.6 μM (P < 0.0001), which corroborated with its previously reported value [29]. Potential antileishmanial activity against *L. major* promastigotes was exhibited by primaquine, suramin and netilmicin with IC_50_ values of 92.9 ± 4.7 μM (P < 0.0001), 90.0 ± 5.0 μM (P < 0.0001), 46.8 ± 2.3 μM (P < 0.0001) respectively.

The drugs primaquine, suramin and netilmicin were next screened against intracellular amastigotes of *L. donovani* and *L. major* strains using RAW 264.7 as host cell. In addition the efficacy of curcumin against intracellular amastigotes was also estimated. In every experiment the percentage of infected MØs ranged from 80 to 95% and the number of amastigotes/100 MØs ranged from 89 to 97.

The EC_50_ values determined for paromomycin, primaquine, netilmicin, suramin and curcumin involving intracellular *L. major* (5ASKH) and *L. donovani* (AG83) amastigotes were 7.5 (± 2.3); 11.8 (0.8); 12.3 (2.3); 4.6 (0.8); 8.1 (1.1) µM and 8.4 (3.3); 6.0 (1.2); 8.6 (1.4); 4.1 (0.3); 12.6 (1.5) µM, respectively (Table 1, in every case P < 0.0001). The value obtained for paromomycin corroborated well with the previously determined report [30]. Best results were obtained for suramin with the lowest EC_50_ value. However, on testing with *L. donovani* parasite (BS13), a rise in EC_50_ values was observed in the case of all the drugs [paromomycin-10.4 ± 1.4 (P < 0.0001); netilmicin-21.1 ± 3.4 (P < 0.0001); suramin-9.1 ± 2.3 (P < 0.0001)] with the exception of curcumin (11.6 ± 2.5; P < 0.0001). In the case of primaquine the drug exhibited suboptimal efficacy up to 40 μm for BS13 (P = 0.0012).

**Table 1:** Susceptibility of intracellular amastigotes of *L. major* and *L. donovani* towards the respective drugs, represented by EC_50_ values

Serial no.	Drug/compound	EC_50_ ± SD (µM)	
		5ASKH^a^	AG83^b^
1	Primaquine	11.8 ± 0.8	6.0 ± 1.2
2	Paromomycin	7.5 ± 2.3	8.4 ± 3.3
3	Netilmicin	12.3 ± 2.3	8.6 ± 1.4
4	Suramin	4.6 ± 0.8	4.1 ± 0.3
5	Curcumin	8.1 ± 1.1	12.6 ± 1.5

Results are given as mean ± SD of three independent experiments

^a^*L. major*; ^b^ *L. donovani*

#### Drug synergism in *Leishmania* promastigotes and amastigotes

Our next step was to estimate synergism between selected drugs using the “modified fixed-ratio isobologram” method. *L. major* promastigotes were initially used to estimate the interactions for all possible drug combinations involving paromomycin, primaquine, netilmicin, and suramin. Consistent synergism was observed only between suramin–paromomycin (Table 2, Additional file 2). For intracellular amastigotes, consistent synergism was observed for the drug combinations suramin–paromomycin (Table 3, Additional file 2, mean $\sum {\text{FICs}}$—*L. major*: 0.34 ± 0.04; *L. donovani*: 0.38 ± 0.07), suramin–netilmicin (Table 3, Additional file 2, 0.40 ± 0.06; 0.41 ± 0.05) and curcumin–netilmicin (Table 3, Additional file 3, 0.23 ± 0.15; 0.35 ± 0.13). For BS13 *Leishmania* parasite, synergism was confirmed for paromomycin–suramin (0.39 ± 0.09) and recorded a relative decline for netilmicin–curcumin (0.51 ± 0.03).

**Table 2:** Assessment of in vitro drug interactions against *L. major* (5ASKH strain) promastigotes

Serial no.	Drug combination	Mean $\sum {\text{FICs}}$^a^	Nature of the interaction
1	Paromomycin–suramin	0.41 ± 0.05	Synergism
2	Netilmicin–suramin	0.69 ± 0.29	Indifference
3	Primaquine–suramin	0.90 ± 0.08	Indifference
4	Primaquine–netilmicin	1.14 ± 0.2	Indifference
5	Primaquine–paromomycin	1.68 ± 0.15	Indifference
6	Paromomycin–netilmicin	2.6 ± 0.4	Indifference

Results are given as mean ± SD of three independent experiments

^a^Mean of $\sum {\text{FICs}}$ were used to define the nature of the interactions between the drugs against *L. major* (5ASKH strain) promastigotes

**Table 3:** Assessment of in vitro drug interactions against intracellular *Leishmania* amastigotes

Drug combination	Mean FIC_S_^a^ Mean $\sum {\text{FICs}}$^a^		Interaction type	
	5ASKH	AG83	5ASKH	AG83
Suramin–paromomycin	0.34 ± .04	0.38 ± 0.07	Synergism	Synergism
Suramin–netilmicin	0.40 ± 0.06	0.41 ± 0.05	Synergism	Synergism
Curcumin–suramin	0.48 ± 0.08	1.18 ± 0.24	Synergism	Indifference
Curcumin–netilmicin	0.23 ± 0.15	0.35 ± 0.13	Synergism	Synergism
Curcumin–paromomycin	0.34 ± 0.15	0.61 ± 0.35	Synergism	Indifference
Curcumin–primaquine	0.63 ± 0.15	0.90 ± 0.38	Indifference	Indifference
Paromomycin–netilmicin	2.1 ± 0.12	1.8 ± 0.18	Indifference	Indifference
Primaquine–paromomycin	1.5 ± 0.2	1.1 ± 0.04	Indifference	Indifference

Results are given as mean ± SD of three independent experiments

^a^Mean of $\sum {\text{FICs}}$ were used to define the nature of the interactions between the drugs against intracellular *L. major* (5ASKH strain) and *L. donovani* (AG83 strain) amastigotes

### Discussion

Given the exorbitant costs in drug development and limited funds available worldwide for neglected tropical diseases, one strategy would be to re-purpose clinically available drugs as anti-leishmanials or utilize abundant natural compounds. Netilmicin (antibiotic) [31], suramin (antitrypanosomatid) and primaquine (antimalarial) [32] are available in the market and curcumin is an abundant natural compound present in turmeric.

Considerable progress has been made in the treatment of VL by the single or suitable co-adminstration of amphotericin B, miltefosine and paromomycin [27]. Paromomycin has been applied as a topical formulation both singly (20–30%) and in combination with gentamicin (0.5%) for the alleviation of CL with approximately 80% curative rates [33–35]. Netilmicin also belongs to the same class of aminoglycoside drugs. Although, netilmicin exhibits antileishmanial action for the *L. major* parasites, animal model studies indicate its reduced efficacy against CL (as a topical application) relative to paromomycin [36]. Again, its efficacy against *L. donovani,* promastigotes were not uniform for all the strains tested in this work. Thus, our work appears to indicate that the most effective action of netilmicin against all forms of the parasite could be in synergistic combination with curcumin.

Suramin has found extensive use in *Trypanosoma brucei rhodesiense* infections causative of African trypanosomiasis and this polysulphonated naphthylamine based compound inhibits glycolytic proteins in the parasite [37]. The antileishmanial efficacy of suramin extends to the promastigote and amastigote stages, exhibiting good efficacy against all the parasitic strains tested in this work.

Synergism tested between various drugs as combination therapy offers several advantages which includes reduced dosage of both drugs, reduced treatment time, less toxicity due to lower dosage and the possibility of delaying the emergence of resistant strains. Reduction in dosage and duration of therapy could lower the financial burden associated with the treatment, increasing its accessibility. Suramin–paromomycin exhibited consistent synergism for all of the *Leishmanial* parasites studied here.

In conclusion, we report the antileishmanial activity of the aminoglycoside netilmicin and the anti-trypanosomatid suramin, with synergism observed between paromomycin–suramin and netilmicin–curcumin (for some strains).

## Limitations

These results have to be confirmed in animal models.

## Additional files

**Additional file 1.** Drug Susceptibility of *Leishmania major* promastigotes towards selected drugs. The drugs triamterene, framycetin, kanamycin, tobramycin, acarbose, gentamicin, lidocaine, primaquine, paromomycin, suramin and netilmicin were screened for their antileishmanial efficacy (IC_50_).

**Additional file 2.** Representative isobolograms of in vitro interactions between the respective drugs. Representative isobolograms for netilmicin–suramin and paromomycin–suramin.

**Additional file 3.** Representative isobolograms of in vitro interactions between the respective drugs. Representative isobolograms for curcumin–netilmicin.

## Abbreviations

CL: cutaneous
MCL: mucocutaneous
VL: visceral
KA: kala-azar
ANOVA: analysis of variance
MØs: murine macrophage
FBS: fetal bovine serum
FICs: fractional inhibitory concentrations
NE: ineffective
