# Supplementary material for: In vitro screening of known drugs identified by scaffold hopping techniques shows promising leishmanicidal activity for suramin and netilmicin
Source: BMC Res Notes. 2018 May 21;11:319. doi: 10.1186/s13104-018-3446-y (PMC5963029; doi:10.1186/s13104-018-3446-y)
Supplement: Supplementary file 1 — Additional file 1. Drug Susceptibility of Leishmania major promastigotes towards selected drugs. The drugs triamterene, framycetin, kanamycin, tobramycin, acarbose, gentamicin, lidocaine, primaquine, paromomycin, suramin and netilmicin were screened for their antileishmanial efficacy (IC50). [file 13104_2018_3446_MOESM1_ESM.pdf]

Additional file 1 Drug Susceptibility of *Leishmania major* (strain 5ASKH) promastigotes towards selected drugs estimated by IC_{50} values. Results are given as mean ± SD of three independent experiments

Serial No.	Drug Name	IC_{50}±SD (µM)
1	Triamterene[a]	NE
2	Acarbose[a]	858±7.2
3	Vidarabine[b]	NE
4	Kanamycin[c]	NE
5	Tobramycin[d]	NE
6	Framycetin[a]	NE
7	Gentamicin[d]	543.1±5.9
8	Lidocaine[a]	NE
9	Primaquine[a]	92.9±4.7
10	Paromomycin[a]	40.8±3.6
11	Suramin[a]	90.0±5
12	Netilmicin[e]	46.8±2.3

NE: Not effective at concentrations in excess of 1500 µM of the respective drugs.
a (all from Sigma-Aldrich Ltd.)
b (from Transport Corporation of India Ltd.)
c (from HiMedia)
d (from Sisco Research Laboratories Pvt. Ltd.)
e (Zuventus Healthcare Ltd.)
