# Supplementary material for: In vitro screening of known drugs identified by scaffold hopping techniques shows promising leishmanicidal activity for suramin and netilmicin
Source: BMC Res Notes. 2018 May 21;11:319. doi: 10.1186/s13104-018-3446-y (PMC5963029; doi:10.1186/s13104-018-3446-y)
Supplement: Supplementary file 2 — Additional file 2. Representative isobolograms of in vitro interactions between the respective drugs. Representative isobolograms for netilmicin–suramin and paromomycin–suramin. [file 13104_2018_3446_MOESM2_ESM.pdf]

Additional file 2

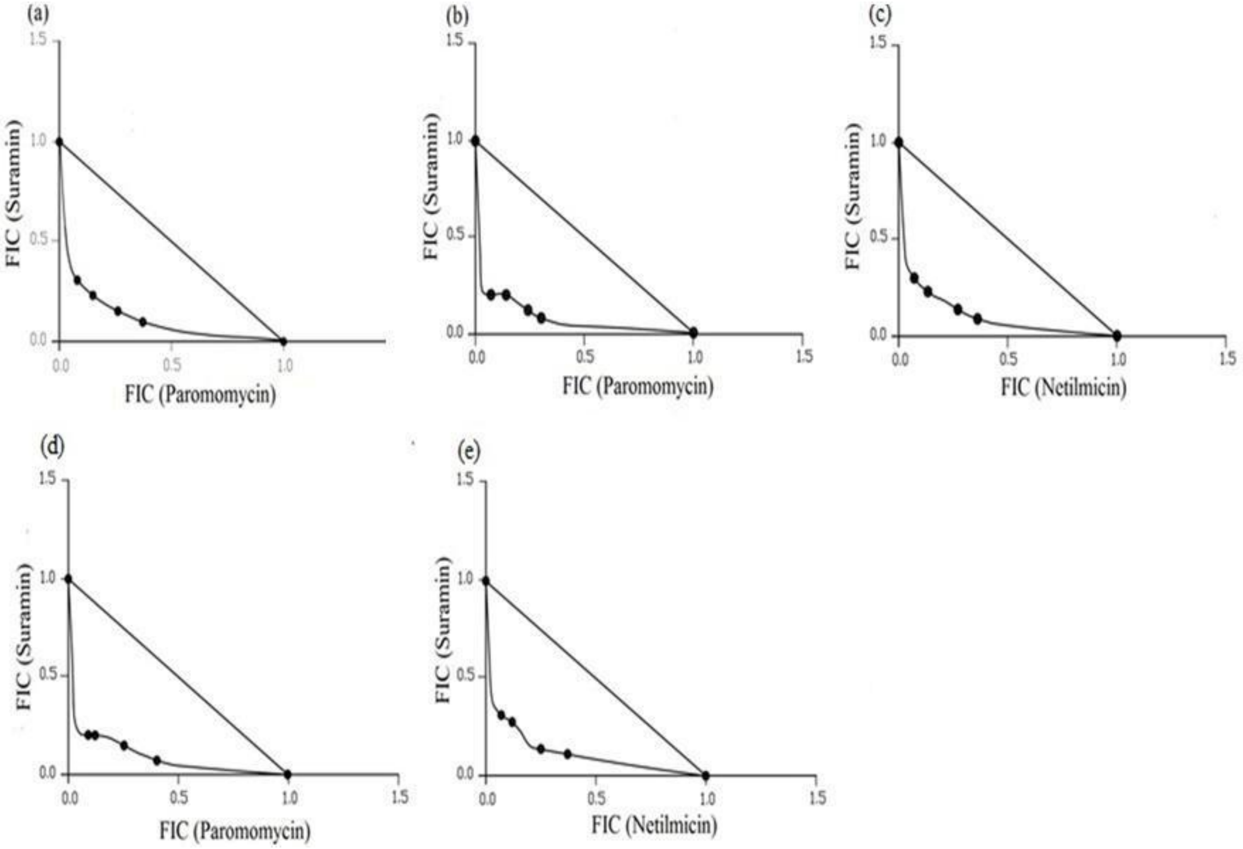

Additional file 2. A representative isobologram of *in vitro* interactions between the respective drugs. (a) paromomycin-suramin against *L. major* promastigotes (5ASKH strain). (b) paromomycin - suramin, (c) netilmicin - suramin against intracellular *L. major* (5ASKH strain) amastigotes and (d) paromomycin – suramin, (e) netilmicin - suramin against intracellular *L. donovani* (AG83 strain) amastigotes. The FICs, calculated and plotted. The bold curve corresponds to the predicted positions of the experimentally determined points for a simple synergistic effect.
