# Supplementary material for: In vitro screening of known drugs identified by scaffold hopping techniques shows promising leishmanicidal activity for suramin and netilmicin
Source: BMC Res Notes. 2018 May 21;11:319. doi: 10.1186/s13104-018-3446-y (PMC5963029; doi:10.1186/s13104-018-3446-y)
Supplement: Supplementary file 3 — Additional file 3. Representative isobolograms of in vitro interactions between the respective drugs. Representative isobolograms for curcumin–netilmicin. [file 13104_2018_3446_MOESM3_ESM.pdf]

Additional file 3

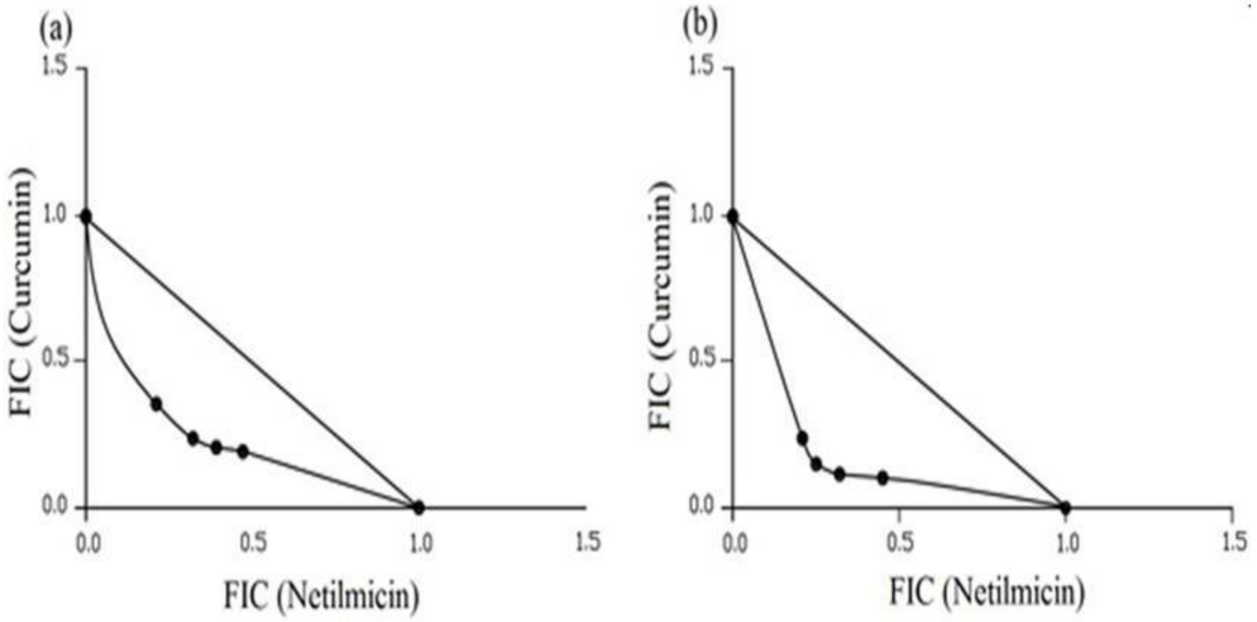

Additional file 3. A representative isobologram of *in vitro* interactions between the respective drugs. curcumin - netilmicin against (a) intracellular *L. donovani* (AG83 strain) amastigotes and (b) intracellular *L. major* (5ASKH strain) amastigotes. The FICs are calculated and plotted. The bold curve corresponds to the predicted positions of the experimental points for a simple synergistic effect.
